# Data Mining Paths for Standard Weekly Training Load in Sub-Elite Young Football Players: A Machine Learning Approach

**DOI:** 10.3390/jfmk9030114

**Published:** 2024-06-28

**Authors:** José E. Teixeira, Samuel Encarnação, Luís Branquinho, Ryland Morgans, Pedro Afonso, João Rocha, Francisco Graça, Tiago M. Barbosa, António M. Monteiro, Ricardo Ferraz, Pedro Forte

**Affiliations:** 1 Department of Sport Sciences, Polytechnic of Guarda, 6300-559 Guarda, Portugal; 2 Department of Sport Sciences, Instituto Politécnico de Bragança, 5300-253 Bragança, Portugal; samuel01.encarnacao@gmail.com (S.E.); barbosa@ipb.pt (T.M.B.); mmonteiro@ipb.pt (A.M.M.); 3 SPRINT—Sport Physical Activity and Health Research & Inovation Center, 6300-559 Guarda, Portugal; joaopdsrocha@gmail.com (J.R.); a.fmgraca7@gmail.com (F.G.); 4 Research Center in Sports, Health and Human Development, 6201-001 Covilhã, Portugal; luisbranquinho@ipportalegre.pt (L.B.); ricardompferraz@gmail.com (R.F.); 5 LiveWell—Research Centre for Active Living and Wellbeing, Polytechnic Institute of Bragança, 5300-253 Bragança, Portugal; 6 CI-ISCE, ISCE Douro, 4560-547 Penafiel, Portugal; 7 Department of Pysical Activity and Sport Sciences, Universidad Autónoma de Madrid, Ciudad Universitaria de Cantoblanco, 28049 Madrid, Spain; 8 Biosciences Higher School of Elvas, Polytechnic Institute of Portalegre, 7300-110 Portalegre, Portugal; 9 Life Quality Research Center (CIEQV), 4560-708 Penafiel, Portugal; 10 School of Sport and Health Sciences, Cardiff Metropolitan University, Cardiff CF23 6XD, UK; 11 Department of Sports, Exercise and Health Sciences, University of Trás-os-Montes e Alto Douro, 5001-801 Vila Real, Portugal; pmvafonso@gmail.com; 12 Department of Sports Sciences, University of Beria Interior, 6201-001 Covilhã, Portugal

**Keywords:** artificial intelligence (AI), periodization, maturation, youth, big data

## Abstract

The aim of this study was to test a machine learning (ML) model to predict high-intensity actions and body impacts during youth football training. Sixty under-15, -17, and -19 sub-elite Portuguese football players were monitored over a 6-week period. External training load data were collected from the target variables of accelerations (ACCs), decelerations (DECs), and dynamic stress load (DSL) using an 18 Hz global positioning system (GPS). Additionally, we monitored the perceived exertion and biological characteristics using total quality recovery (TQR), rating of perceived exertion (RPE), session RPE (sRPE), chronological age, maturation offset (MO), and age at peak height velocity (APHV). The ML model was computed by a feature selection process with a linear regression forecast and bootstrap method. The predictive analysis revealed that the players’ MO demonstrated varying degrees of effectiveness in predicting their DEC and ACC across different ranges of IQR. After predictive analysis, the following performance values were observed: DEC ($\bar{x}$_predicted_ = 41, β = 3.24, intercept = 37.0), lower IQR (IQR_predicted_ = 36.6, β = 3.24, intercept = 37.0), and upper IQR (IQR_predicted_ = 46 decelerations, β = 3.24, intercept = 37.0). The player’s MO also demonstrated the ability to predict their upper IQR (IQR_predicted_ = 51, β = 3.8, intercept = 40.62), lower IQR (IQR_predicted_ = 40, β = 3.8, intercept = 40.62), and ACC ($\bar{x}$_predicted_ = 46 accelerations, β = 3.8, intercept = 40.62). The ML model showed poor performance in predicting the players’ ACC and DEC using MO (MSE = 2.47–4.76; RMSE = 1.57–2.18: R^2^ = −0.78–0.02). Maturational concerns are prevalent in football performance and should be regularly checked, as the current ML model treated MO as the sole variable for ACC, DEC, and DSL. Applying ML models to assess automated tracking data can be an effective strategy, particularly in the context of forecasting peak ACC, DEC, and bodily effects in sub-elite youth football training.

## 1. Introduction

In youth football, the application of artificial intelligence (AI) and machine learning (ML) methods has emerged as a game-changer for long-term player development, performance analysis, and injury prevention [1,2]. Understanding the factors influencing the long-term development, trainability, and individual performance of young footballers has been described as one of the greatest applications of ML methods, making it possible to automate data collection and subsequent application [3]. In fact, ML aids in deciphering intricate patterns within vast datasets, shedding light on the interplay of talent identification predictors, match-related contextual factors and training regimes in shaping future footballing prowess [4,5]. Supervised and unsupervised ML methods serve different purposes in data analysis and prediction. Supervised learning relies on labeled data to train models, making it ideal for tasks where outcomes are known and predictions are required [6,7]. Within supervised learning, regression methods are pivotal for predicting continuous outcomes based on input features. Unsupervised learning, on the other hand, does not use labeled data and is often used for clustering and pattern discovery. Both supervised and unsupervised techniques are integral to ML models, each serving unique functions depending on the problem at hand [7]. Also, ML algorithms excel in discerning subtle nuances between elite and sub-elite contexts, offering invaluable insights into performance differentials [8,9]. By analyzing diverse metrics encompassing technical proficiency, tactical acumen, and physical attributes, these algorithms pave the way for targeted interventions and talent identification [10]. The advent of wearable technology and tracking systems has heralded new insights in training load monitoring [11,12]. ML algorithms leverage real-time data streams from these devices to quantify physiological stress, optimize training protocols, and mitigate injury risks, thereby enhancing player welfare and performance sustainability [13].

Drawing upon extensive literature, ML facilitates the identification of optimal training loads tailored to the developmental needs of young footballers. By integrating age-specific physiological parameters, growth trajectories, and injury prevalence rates, these models strive to strike a delicate balance between maximizing performance gains and safeguarding long-term health [14]. The relationship between technical, tactical, and physical facets underscores the holistic nature of footballing excellence [15,16]. ML algorithms elucidate the intricate interdependencies between these dimensions, fostering a comprehensive understanding of player development trajectories and strategic gameplay evolution [1,3]. Successful offensive and defensive actions are dependent on peak accelerations (ACCs), decelerations (DECs), and body impacts; however, biological maturation can greatly influence individual performance and competition outcomes in youth football [8,17].

From neural networks and decision trees to support vector machines and deep learning architectures, the arsenal of ML models is vast and diverse. Each model offers unique capabilities suited to distinct footballing applications, ranging from player performance prediction to opponent scouting and tactical optimization [18,19]. In the footballing realm, ML finds several applications, ranging from injury prediction and prevention to talent identification, performance analysis, and tactical insights [20,21] by harnessing the power of big data analytics and advanced algorithms, enhanced decision-making and strategic nuances [22]. As the nexus between technology and athleticism continues to evolve, the synergy between AI, ML, and network analysis in football promises to reshape the sporting landscape, unlocking new frontiers of innovation and excellence for predicting physical performance [23,24]. While unsupervised learning is typically used to comprehend relationships among datasets, supervised ML is typically used to classify data or create predictions [25]. Because tagged data are required, supervised ML requires far more resources [26]. Thus, the aim of this study was to test an unsupervised ML model to predict high-intensity actions and body impacts during youth football training, specifically in under-15, -17, and -19 sub-elite Portuguese football.

## 2. Materials and Methods

### 2.1. Sample

Sixty male youth football players were subjected to monitoring over a 2-week interval within a sub-elite Portuguese football academy setting. The cohort comprised twenty players each from the under-15 (U15), under-17 (U17), and under-19 (U19) age categories. For the U15 group, the mean age was 13.2 ± 0.5 years, with corresponding mean height and weight values of 1.69 ± 0.78 m and 55.7 ± 9.4 kg, respectively. The U17 cohort exhibited a mean age of 15.4 ± 0.5 years, mean height of 1.8 ± 0.5 m, and mean weight of 64.38 ± 6.6 kg. Likewise, the U19 players demonstrated a mean age of 17.39 ± 0.55 years, mean height of 1.82 ± 0.01 m, and mean weight of 68.9 ± 8.4 kg. Overall, the average height was 1.74 ± 0.08 m, weight 62.48 ± 10.03 kg, body mass index (BMI) 20.61 ± 2.14 kg/m^2^, sitting height 88.36 ± 8.51 cm, predicted adult height 14.20 ± 1.39 cm, average experience 6.76 ± 1.42 years, and relative age 0.25 ± 0.18. All participants received comprehensive information regarding the purpose and potential risks of the research according to ethical standards. The study protocol was approved by the local Ethics Committee at the University of Trás-os-Montes e Alto Douro (3379-5002PA67807).

### 2.2. Procedures

The young sub-elite football players underwent monitoring throughout training sessions utilizing a portable GPS system (STATSports Apex^®^, Newry, Northern Ireland). This GPS device recorded raw position, velocity, and distance data at sampling frequencies of 18 Hz, complemented by an accelerometer (100 Hz), magnetometer (10 Hz), and gyroscope (100 Hz) [27,28]. Each player wore the device within a micro-tech inner mini pocket embedded in a custom-made vest positioned on the upper back between the shoulder blades. Activation of all devices occurred 30 min prior to the commencement of training sessions to ensure optimal satellite signal reception. A minimum of eight available satellite signals was deemed necessary to maintain optimal signal strength for accurate human movement measurement [29]. The current GPS dataset should account for a small margin of error, approximately 1–2% with an ideal horizontal dilution of precision (HDOP) of 0.4, as reported in the 10–15 Hz STATSports Apex^®^ units [27]. Perceived exertion was assessed utilizing the 15-point Borg Rating of Perceived Exertion 6–20 Scale (Borg RPE 6–20) [30]. The session rating of perceived exertion (sRPE) was calculated by multiplying each individual’s RPE score by the total duration of the training session (sRPE = RPE × session duration), with scores ranging from 6 to 20 [31]. Additionally, to gauge recovery status, players provided total quality recovery (TQR) scores on a scale from 6 to 20, as proposed by Kenttä and Hassmén [32] to capture athletes’ perceptions of recovery. Both RPE and TQR scores were collected individually approximately 30 min before and after each training session, respectively. Players were familiar with the assessment procedures, and perceived data were recorded using a Microsoft Excel^®^ spreadsheet (version 16.46, Microsoft Corporation, Redmond, WA, USA). Prior studies have employed both scales to investigate perceived stress and fatigue levels in youth football contexts [33,34].

### 2.3. Target Variables

We selected the external training load (ETL), dynamic stress load (DSL), number of accelerations (ACCs), and number of decelerations (DECs) as the target variables. The acceleration variables, ACCs and DECs, took into account movements inside the maximal intensity zone, which is defined as >3 m/s and <3 m/s, respectively [13]. A 100 Hz tri-axial accelerometer built into the GPS devices measured the accelerations in the X, Y, and Z planes, three orthogonal axes of movement, to assess DSL and produce a composite magnitude vector (represented as G force): [(*a*_*y*1_ − *a*_*y*−1_)^2^ + (*a*_*x*1_ − *a*_*x*−1_)^2^ + (*a*_*z*1_ − *a*_*z*−1_)^2^], where *a_x_* = mediolateral acceleration, *a_y_* = anteroposterior acceleration, and *a_z_* = vertical acceleration. The DSL was expressed in arbitrary units (a.u.) [35]. In addition, we selected maturation offset (MO), age at peak height velocity (APHV), and chronological age. As previously established for youth team sports [36,37], maturity status was determined using a predictive Mirwald’s equation that took into account chronological age, standing height, sitting height, and body mass [38]. Maturation time, also known as age at peak height velocity (PHV), is the age at which a particular maturational event occurs [39,40]. Z scores were used to determine maturity timing: more than 0.5 indicated early status; between −0.5 and +0.5 indicated average maturity timing, indicating that the athletes were regarded as average in their maturity phases; and less than −0.5 indicated late maturity timing [41,42]. The rating of perceived exertion (RPE), session RPE (sRPE), and total quality recovery (TQR) were used as the predictive perceived exertion [8,9]. Other ETL-based measures were excluded: total distance (TD) covered (m), average speed (AvS), maximal running speed (MRS) (ms^−1^), relative high-speed running (rHSR) distance (m), high metabolic load distance (HMLD) (m), and sprinting (SPR) distance (m).

### 2.4. Data Preprocessing

Prior to applying ML models, we performed a featuring selection analysis with an interpolation strategy to identify the features most related to the players’ MO. For this purpose, we chose a correlation matrix as the desired method [43]. Next, we found the most important features in ACC (r = 0.30) and DEC (r = 0.24), detailed in Section 3. Thus, we arranged the features ACC and DEC into an X array and the target variables into a y array. After this, to equalize feature scales, we performed data normalization in the X array, where the features were converted in a scale with range from −1.1. Using the function “train_test_split”, we split 70% of the X and y arrays to train and 30% to test the algorithms’ predictions, with a random state = 42 to guarantee the same aleatorization seed for all algorithms during training and testing tasks [44]. Data are presented as linear regression (β) and average ($\bar{x}$), upper (75%), and lower (25%) interquartile range (IQR). The correlation magnitude was classified as: trivial if r ≤ 0.1, small if r = 0.1–0.3, moderate if r = 0.3–0.5, large if r = 0.5–0.7, very large if r = 0.7–0.9, and almost perfect if r ≥ 0.9 [42,43].

### 2.5. Data Analysis

After this, from the libray “sklearn” [43], we applied seven ML regressive algorithms: extreme gradient boosting regression (XGboost) with the function “from xgboost import XGBRegressor”, Bayesian regresison (BR), with the function “from sklearn.linear_model import BayesianRidge”, linear regression (LR) with the function “from sklearn.linear_model import LinearRegression”, ridge regression (RR) with the function “from sklearn.linear_model import Ridge”, decision tree (DT) regression with the function “from sklearn.tree import DecisionTreeRegressor”, random forest (RF) regression activating the function “from sklearn.ensemble import RandomForestRegressor”, and support vector regression (SVR) with the function “from sklearn.svm import SVR”. Also from the “sklearn” library, we activated the functions “from sklearn.metrics import mean_squared_error, r2_score” to calculate mean square error (MSE), root mean square error (RMSE), and coefficient of determination (R^2^) [45,46].

## 3. Results

### 3.1. Feature Selection

**Figure 1:**
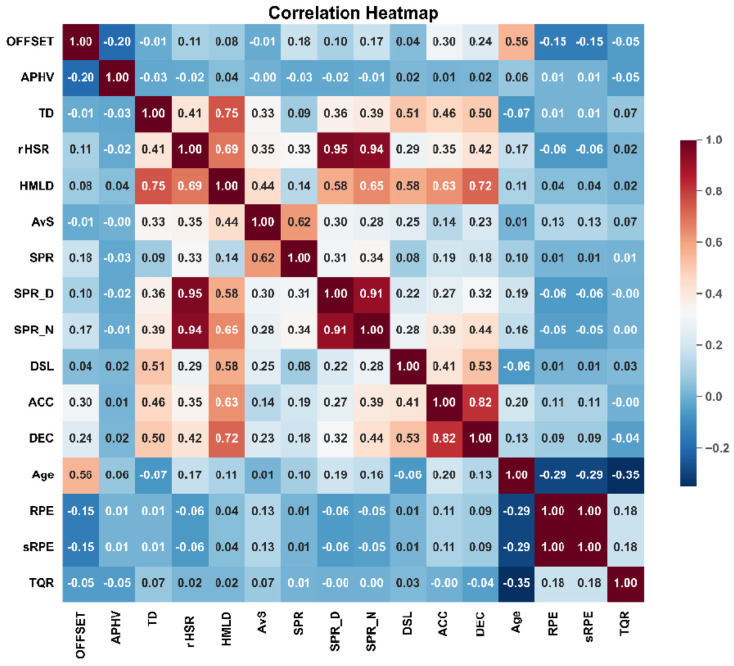
Full dataset of correlation coefficients. Note: ACCs—number of accelerations; APHV—age at peak height velocity; AvS—average speed; DECs—number of decelerations; DSL—dynamic stress load; HMLD—high metabolic load distance; OFFSET—maturation offset; rHSR—relative high-speed running distance; SPR—sprinting distance; TD—total distance covered.

The players’ MO emerged as the foremost contributor to the prediction of both deceleration (DEC) and acceleration (ACC) metrics, with correlation coefficients of r = 0.24 and r = 0.30, respectively, indicative of moderate effect sizes (ESs) (Figure 1). Conversely, despite rigorous analysis, the feature selection model failed to identify any significant characteristics associated with the DLS prediction for the players. Figure 1 provides the correlation coefficients observed across the dataset, showing relationships between various predictor variables and match running-based performance metrics.

### 3.2. Data Interpolation

**Figure 2:**
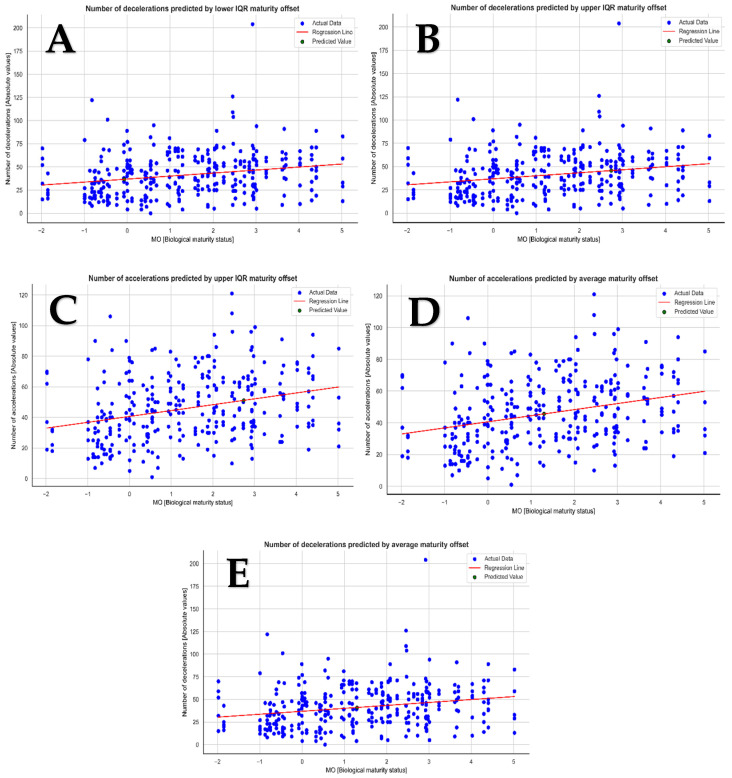
Linear regression forecast: (**A**) players’ DEC prediction by their lower IQR MO; (**B**) players’ DEC prediction by their upper IQR MO; (**C**) players’ ACC prediction by their lower IQR MO; (**D**) players’ ACC prediction by their upper IQR MO; (**E**) players’ DEC prediction by their average. Note: the blue dots represent the current date, the red line the regression, and the black dots the predicted values.

Additionally, using an ML algorithm, we created a linear regression strategy to predict the football players’ ACC and DEC based on average ($\bar{x}$), upper (75%), and lower (25%) interquartile range (IQR). The performance values observed in predicting the players’ DEC were ($\bar{x}$_predicted_ = 41, β = 3.24, intercept = 37.0), lower IQR (IQR_predicted_ = 36.6, β = 3.24, intercept = 37.0), and upper IQR (IQR_predicted_ = 46, decelerations, β = 3.24, intercept = 37.0) after predictive analysis. The players’ MO also demonstrated the ability to predict their upper IQR (IQR_predicted_ = 51, β = 3.8, intercept = 40.62), lower IQR (IQR_predicted_ = 40, β = 3.8, intercept = 40.62), and ACC ($\bar{x}$_predicted_ = 46 accelerations, β = 3.8, intercept = 40.62). The upper IQR MO prediction for each target GPS variable in the ML model is displayed in Figure 2.

### 3.3. Machine Learning Results

Table 1 expresses the results from the seven ML algorithms implemented. All showed poor performance in predicting the players’ ACC and DEC using MO values, as proved by the high MSE (2.47 to 4.76) and RMSE (1.57 to 2.18) and low R^2^ values (−0.78 to 0.02).

## 4. Discussion

The use of ML models to assess automated tracking data is intriguing, particularly in the context of forecasting peak ACC, DEC, and bodily effects in young sub-elite football training. The findings of this study highlight the potential of using ML approaches, particularly linear regression, to predict high-demand actions and body impacts in sub-elite youth football. The correlation analysis revealed moderate ES for both ACC and DEC, indicating a meaningful relationship between these metrics and player performance. However, despite these correlations, traditional feature models failed to identify significant characteristics for predicting the distance covered at sprinting speed (DSL), suggesting the complexity and multifactorial nature of this particular training load metric.

The utilization of ML algorithms allowed for a more nuanced approach to prediction, leveraging not only average values but also the variability within the dataset, as represented by the IQR scores. By incorporating both the mean and the upper/lower IQR, the linear regression model demonstrated promising performance in predicting DEC and ACC. Notably, the consistent β coefficients across different quartiles and intercepts underscore the stability and reliability of the predictive model. Logical regression and conditional random fields were used to analyze spatiotemporal patterns leading up to shots, effectively quantifying shot efficiency and team strategy [47,48]. By scrutinizing a full season’s worth of tracking data, they provided insights into the impact of strategic features on the likelihood of scoring goals. This study not only advances the understanding of football analytics but also offers practical implications for coaches and analysts seeking to optimize team performance through data-driven strategies [49,50]. It is possible that training tasks designs like small-sided games (SSG) [31,36], speed, agility, and quickness (SAQ) [13], and repeated sprint ability (RSA) [51,52] can have a substantial impact on this ACC and DEC performance. Also, all ML regressive algorithms showed poor performance in predicting the players’ ACC and DEC using MO. This development is more dependent on the training tasks’ constraints and the playing environment than biological maturity.

Moreover, the inclusion of the players’ movement orientation as a predictor variable should further enhance the predictive accuracy, particularly for upper and lower IQR values of DEC and ACC [53]. This suggests that not only the raw metrics of acceleration and deceleration but also the directionality of movement, which play a crucial role in performance prediction. The MO-based predictions exhibited higher values for upper IQR, indicating that players with certain movement orientations tend to exhibit greater variability in their performance metrics. Previously, an interaction effect was observed among chronological age, relative age, and maturation concerning accumulated training load in young sub-elite football players [8,54]. However, perceived exertion did not exhibit notable differences across age groups or maturity status. Notably, the within–between interaction analysis revealed significant differences in all variables when comparing age groups and maturation status [54]. These findings offer valuable insights for coaches and sports scientists in prescribing and controlling training loads tailored to the specific needs of young football players, thereby optimizing their development and performance trajectories.

The visualization of the upper IQR predictions for each target GPS variable in the ML model provides valuable insights into the variability and distribution of performance metrics among football players [55,56]. This visualization can aid coaches and sports scientists in identifying outliers, understanding patterns of performance, and tailoring training programs to address specific strengths and weaknesses [16,57]. Future research should also consider the importance of certain fundamental points in motor skills, tactical knowledge, kinesthetic differentiation, and spatial orientation in the development of technical and physical actions and their interdependence with the physical and biological component [33,58,59]. The perception of motor competence and physical and emotional well-being are preponderant in physical performance, and monitoring strategies should also include means to monitor mental fatigue [58].

Overall, this study showcases the potential of ML techniques, specifically linear regression incorporating IQR and MO, in predicting key performance metrics in football players. However, further research is warranted to validate these findings across diverse player populations and to explore additional predictor variables that may enhance the predictive accuracy of the model. Additionally, longitudinal studies could provide insights into the stability of these predictive models over time and their utility in informing player development and performance optimization strategies. Future ML models should contextualize the actions of ACC, DEC, and DSL for both individual and group tactical behavior in training tasks. Recently, studies developed ML approaches to estimate scoring opportunities in football matches by analyzing strategic features extracted from player and ball-tracking data [55,56]. Those studies employed logistic regression and conditional random fields to scrutinize spatiotemporal patterns preceding shots, thereby quantifying shot efficiency [60,61] and team strategy [62,63]. The findings underscored the significance of strategic features in influencing the likelihood of scoring goals, providing valuable insights for coaches and analysts seeking to optimize team performance through data-driven strategies [64,65]. Additionally, Chawla et al. [66] contributed to the field by introducing an automated system for evaluating passes in football matches using trajectory data and computational geometry [67,68]. Their ML-driven approach achieved high accuracy in pass rating, facilitated by the incorporation of complex data structures derived from computational geometry [69,70]. Other research not only showcased the potential of advanced analytics in football but also highlighted the efficacy of interdisciplinary approaches, merging concepts from geometry with ML techniques to enhance performance analysis in sports [3]. Beyond the prediction, a team’s entropy score plays a significant role in predicting players’ and teams’ positions during a season.

Mental and psychological health issues determine certain psychophysiological conditions that influence physical performance [57,59]. Currently, predictive models of the influence of psychophysiological variables are used for training load and match running performance [16,57]. In the area of the relationship between mental and psychological health and physical performance, several studies have been conducted to understand how psychophysiological conditions influence athletic performance [71,72]. Some of the main studies in this area include research investigating anxiety and stress as factors affecting decision-making during the game, motivation as a driver of physical performance, and self-confidence as a predictor of sports success [73,74]. For example, research has shown that elevated levels of anxiety can impair athletes’ attention and concentration, negatively impacting performance during competitions. Similarly, excessive stress can lead to mental and physical fatigue, decreasing reaction capacity and the effectiveness of movements during the game [59,75,76]. Conversely, studies have shown that highly motivated athletes tend to persist longer in intense physical activities and recover more quickly from injuries [75,77,78]. Additionally, self-confidence has been associated with greater assertiveness in decision-making during the game and better technical execution in various sports. These studies have significantly contributed to the development of predictive models seeking to understand and predict how psychophysiological variables influence training load and performance during matches [78]. However, there is still a research gap to explore this relationship between psychophysiological variables and technical, tactical, and physical performance [57]. Also, in youth football contexts ML-based methodologies in injury prevention and athletic development continue to be applied [7].

## 5. Conclusions

The use of ML algorithm models to assess automated tracking data is intriguing, particularly in the context of forecasting peak ACC, DEC, and bodily effects in sub-elite young football training. In training challenges, future ML models ought to contextualize the behaviors of ACC, DEC, and DSL in terms of both individual and collective tactical behavior.

## Figures and Tables

**Table 1:** Machine learning regressive algorithms.

Algorithm	MSE	RMSE	R^2^
Extreme boosting regression (XGboost)	3.52	1.87	−0.32
Bayesian regression (BR)	2.58	1.60	0.02
Linear regression (LR)	2.57	1.60	0.034
Ridge regression (RR)	2.57	1.60	0.034
Decision tree (DT) regression	4.76	2.18	−0.78
Random forest (RF) regression	2.77	1.66	−0.04
Support vector regression (VMR)	2.47	1.57	0.07

Note. MSE—mean square error; RMSE—root mean square error; R^2^—coefficient of determination. BR—Bayesian regression; DT—Decision tree regression; LR—Linear regression; RF—Random forest regression; RR—Ridge regression; VMR—Support vector regression; XGboost—Extreme boosting regression.

## Data Availability

Data are available upon request to the contact author.
